# Incidence, Predictors, Causes, and Cost of 30-Day Hospital Readmission in Chronic Obstructive Pulmonary Disease Patients Undergoing Bronchoscopy

**DOI:** 10.7759/cureus.8607

**Published:** 2020-06-13

**Authors:** Oday Z AlHafidh, Jasdeep S Sidhu, Jeevanjot Virk, Neel Patel, Zeel Patel, Vijay Gayam, Dina Altuhafy, Osama Mukhtar, Ramakanth Pata, Binav Shrestha, Joseph Quist, Danilo Enriquez, Frances Schmidt

**Affiliations:** 1 Pulmonary, Interfaith Medical Center, Brooklyn, USA; 2 Internal Medicine, Interfaith Medical Center, Brooklyn, USA; 3 Internal Medicine, Ahmedabad Municipal Corporation Medical Education Trust Medical College, Ahmedabad, IND; 4 Internal Medicine/Pulmonology, Interfaith Medical Center, Brooklyn, USA; 5 Pulmonary Medicine, Interfaith Medical Center, Brooklyn, USA

**Keywords:** copd, length of stay, bronchoscopy, readmission, index admission (ia)

## Abstract

Introduction

Chronic obstructive pulmonary disease (COPD) has a significant disease burden and is among the leading causes of hospital readmissions, adding a significant burden on healthcare resources. The association between 30-day readmission in a COPD patient undergoing bronchoscopy and a wide range of modifiable potential risk factors, after adjusting for sociodemographic and clinical factors, has been assessed, and comparison has been made with COPD patients not undergoing bronchoscopy.

Methods

We conducted a comprehensive analysis of the 2016 Nationwide Readmission Database (NRD) of 30-day all-cause readmission among COPD patients undergoing bronchoscopy. A Cox’s proportional hazards model was used to obtain independent relative risks of readmission following bronchoscopy in COPD patients as compared to patients not undergoing bronchoscopy. Our primary outcome was the 30-day all-cause readmission rate in both groups. Other secondary outcomes of interest were the 10 most common reasons for readmission, resource utilization, independent predictors of readmission, and relative proportion of comorbidities between the index admission (IA) and the readmission in both groups.

Results

The overall rate of readmission following bronchoscopy in COPD patients as compared to patients not undergoing bronchoscopy was 17.32% and 15.87%, respectively. The final multivariate model in the bronchoscopy group showed that the variables found to be an independent predictor of readmission were: pulmonary hypertension (hazard ratio [HR] 2.35; 95% confidence interval [CI] 1.26-4.25; P < .01), adrenal insufficiency (HR 4.47; 95% CI 1.44-13.85; P = .01) and discharge to rehab status. Independent predictor variables of admission in Group B were gender (women < men; HR 0.91; 95% CI 0.88-0.93; P < .01), and type of insurance (Medicaid > Medicare > private insurance).

For all patients undergoing bronchoscopy, the mean length of stay (LOS) for IA was 11.91 ± 20.21 days, and LOS for readmission was 5.87 ± 5.48 days. The mean total cost of IA for patients undergoing bronchoscopy was much higher than that of readmission ($26,916 vs. $12,374, respectively). The entire LOS for readmission was 1,265 days, with a total cost of $2.66 million.

For patients not undergoing bronchoscopy during the IA, mean LOS for IA was 4.26 ± 4.27 days, and mean LOS for readmission was 5.39 ± 5.51 days, which was longer than the IA in Group B but still shorter than LOS for readmission in Group A (patients undergoing bronchoscopy). The mean total cost of readmission was higher than the IA ($8,137 for IA vs. $10,893 for readmission). The total LOS in this group of patients was 313,287 days, with the total cost of readmission at $628 million.

Conclusions

Patients undergoing bronchoscopy have a slightly higher rate of 30-day readmissions as compared to patients not undergoing bronchoscopy, and the LOS is also slightly higher in this group during subsequent readmissions as compared to readmission in patients not undergoing bronchoscopy in IA. The readmission rate in COPD patients is impacted by a variety of social, personal, and medical factors. Patients with multiple medical comorbidities have a higher risk of readmission. In our understanding, bronchoscopy in a patient with acute exacerbation of COPD should be reserved for selected patients, and the rationale should be clarified, as it affects the overall LOS and healthcare expenditure.

## Introduction

Chronic obstructive pulmonary disease (COPD) is a leading cause of global morbidity and disability. COPD has become the third highest cause of death worldwide in 2020 [1]. Medical treatment options are limited, and most only provide symptomatic improvements without mortality benefit. The only interventions found to have a mortality benefit in severe COPD are supplemental oxygen for hypoxic patients and pulmonary rehabilitation [2]. Usually, the risk of recurrent exacerbations is high within the first few weeks of index admissions (IAs) [3]. These exacerbations are associated with impaired quality of life, reduced survival, and high healthcare expenditure.

COPD patients have a variety of indications to undergo bronchoscopy. Typically, these patients have a history of exposure to cigarette smoking, thereby putting them in a major risk group for malignancy and infection [4,5]. The main reason for bronchoscopy in patients without COPD is a pulmonary infection rather than suspicion of malignancy (as with patients with COPD) [1]. Recently, interventional bronchoscopy has grown as a field and as a treatment option for the disease itself (e.g., bronchoscopic lung volume reduction [LVR]) and its comorbidities (e.g., laser and stenting placement). Similarly, minimally invasive therapies using bronchoscopy are actively being studied with the ultimate goal of reproducing the advantages seen in surgical LVR with less risk and morbidity from the procedure. Some of the reported bronchoscopic techniques include unidirectional bronchial valves, biologic and polymer-based techniques to occlude lung segments, small stents placed through airway walls to allow trapped air to exist (airway bypass stents), thermal/steam vapor ablation, and endobronchial coils.

The knowledge about which factors relate to COPD exacerbations or hospital admissions for exacerbations is currently very limited. In this study, we tried to identify the incidence, predictors, causes, and costs of 30-day readmission in COPD patients with and without bronchoscopy using a nationally representative database in the United States.

## Materials and methods

Baseline patient characteristics included in our study were age, sex, insurance status (Medicare, Medicaid, private and uninsured), median household income for a patient’s zip code ($1-$38,999; $39,000-$47,999; $48,000-$62,999; $63,000 or more), patient residence (large metropolitan areas with at least one million residents, small metropolitan areas with fewer than one million residents, micropolitan areas [non-urban residential], and not metropolitan or micropolitan), hospital size based on the number of beds (small, medium, and large), hospital volume (expressed in quintiles), and teaching status.

The principal diagnoses and comorbidities such as atrial fibrillation, acute kidney injury (AKI), respiratory complications, anemia, mechanical ventilation, coronary artery disease (CAD) and CAD equivalents, smoking, prior stroke, prior myocardial infarction, gastroesophageal reflux disease (GERD), pulmonary hypertension, hypertension (HTN), obesity, dyslipidemia, peripheral vascular disease, chronic lung disease, diabetes mellitus (DM), congestive heart failure (CHF), chronic kidney disease (CKD), sinusitis, flu, and adrenal insufficiency are identified with their appropriate International Statistical Classification of Diseases-10 (ICD-10) codes. The comorbidity burden was assessed by Deyo’s modification of the Charlson comorbidity index (CCI).

We divided total patients into two groups depending on whether the patients had undergone bronchoscopy during IA or not. We assessed the most common causes of readmission following discharge (e.g., acute COPD exacerbation, pneumonia, septicemia, acute or chronic respiratory failure, acute asthma exacerbation, acute bronchitis with COPD, acute respiratory failure, acute on chronic diastolic and systolic heart failure, orthostatic hypotension, fluid overload, abdominal pain, atrial fibrillation, tracheostomy complications) with the appropriate ICD-10 codes.

Time to readmission was the number of days from discharge (day 0) to the first readmission up to day 30.

The most common diagnosis of readmission was calculated by tallying the primary diagnosis with that of the readmitted patient.

Our primary outcome of interest was 30-day all-cause of readmission for patients who underwent bronchoscopy in 2016. Readmission was identified following the methodology described under the US Healthcare Cost and Utilization Project. We documented only the first readmission if the patient admitted multiple times within 30 days. We excluded admission that was transferred to another hospital and readmitted due to a traumatic cause. Both urgent and elective readmission were included in the analysis. The patients who died in the IA were excluded from the denominator.

The secondary outcomes of interest were (1) the 10 most common reasons for readmission and their relative percentage for bronchoscopy, (2) resource utilization associated with the readmission (LOS, total hospitalization cost, and charges), (3) independent predictors of readmission, and (4) relative proportion of comorbidities between the IA and the readmission in both groups.

Statistical analysis was performed with the use of STATA software, version 15.0 (Stata Corp, College Station, TX). Univariate Cox regression analysis was used to calculate the unadjusted odds ratio for the primary and secondary outcomes. Subsequently, multiple confounders were used to build in the model for a multivariate Cox regression analysis model after running them in univariate regression analysis and taking only those confounders expected to affect the outcome with a P-value less than .2. Calculation of proportions was done using the Fisher exact test, and continuous variables were compared using the Student t-test. All P values of the tests were two-sided, and P-value less than .05 was taken as the threshold for statistical significance.

## Results

We reported baseline patient and hospital characteristics and comorbidities for patients with index hospitalization in Table 1.

**Table 1:** Patient demographics Abbreviations: AKI, acute kidney injury; CAD, coronary artery disease; CHF, congestive heart failure; CKD, chronic kidney disease; COPD, chronic obstructive pulmonary disease; GERD, gastroesophageal reflux disease; MI, myocardial infarction; PVD, peripheral vascular disease

Variable	COPD and Bronchoscopy	COPD without Bronchoscopy
Total	N = 1,358	N = 365,817
Women %	56.46 (766)	55.31 (202,333)
Mean age in years	67.6 ± 12.0	68.5 ± 12.0
Insurance provider %		
Medicare	76.59 (1,040)	74.26 (271,656)
Medicaid	10.59 (145)	12.48 (45,653)
Private	11.75 (159)	10.43 (38,155)
No insurance	1.06 (14)	2.82 (10,353)
Charlson comorbidity index %		
0	0 (0)	0 (0)
1	45.49 (618)	39.95 (146,143)
2	21.74 (295)	26.37 (96,466)
3 or more	32.77 (445)	33.68 (123,208)
Median income in patient zip code %		
$1-$38,999	34.48 (469)	36.15 (132,243)
$39,000-$47,999	31.06 (423)	29.39 (107,514)
$48,000-$62,999	22.43 (303)	19.79 (72,395)
≥ $63,000	12.02 (163)	14.67 (53,665)
Patient residence%		
Large metropolitan areas with at least one million residents	33.26 (452)	45.05 (164,801)
Small metropolitan areas with less than one million residents	61.28 (832)	34.18 (125,036)
Micropolitan areas + non-urban residual	5.46 (74)	20.77 (75,980)
Bed size		
Small	15.49 (210)	21.97 (80,370)
Medium	26.88 (365)	29.75 (108,831)
Large	57.63 (783)	48.28 (176,616)
Teaching hospital	55.54 (754)	46.89 (171,531)
Non-teaching hospital	44.46 (604)	53.11 (194,286)
Urban hospital location	61.00 (828)	67.67 (247,548)
Non-urban hospital location	39.00 (530)	32.33 (118,269)
Hospital volume quintile		
1	6.34 (86)	1.90 (6,951)
2	7.61 (103)	6.26 (22,900)
3	10.50 (143)	13.02 (47,629)
4	16.32 (222)	23.11 (84,540)
5	59.22 (804)	55.71 (203,797)
Atrial fibrillation	18.76 (255)	16.83 (61,567)
AKI	13.41 (182)	8.26 (30,216)
Respiratory complications	20.50 (278)	8.32 (30,436)
Anemia	5.40 (73)	3.29 (12,035)
Mechanical ventilation	1.68 (23)	0.75 (2,744)
CAD and CAD equivalent	22.65 (308)	22.72 (83,114)
Smoking	34.63 (470)	35.73 (130,706)
Prior stroke	5.90 (80)	6.29 (23,010)
Prior MI	5.77 (78)	7.44 (27,217)
GERD	25.40 (345)	22.60 (83,772)
Pulmonary Hypertension	11.56 (157)	7.98 (29,192)
Hypertension	51.00 (693)	54.27 (198,529)
Obesity	19.36 (263)	15.91 (58,201)
Dyslipidemia	41.21 (560)	37.81 (138,315)
PVD	5.44 (74)	6.36 (23,266)
Chronic lung disease	19.31 (262)	12.59 (46,056)
Diabetes	27.78 (377)	29.47 (107,806)
CHF	25.48 (346)	28.33 (103,635)
CKD	13.27 (180)	14.28 (52,238)
Sinusitis	0.49 (7)	0.32 (1,171)
Flu	0.85 (12)	0.81 (2,963)
Adrenal insufficiency	1.07 (15)	0.25 (915)
Discharge to rehab	0.95 (13)	0.42 (1,536)

The mean age of patients undergoing bronchoscopy (Group A) was 67.6 ± 12.0 years, with 56% women. The mean age of COPD patients not undergoing bronchoscopy (Group B) was 68.5 ± 12.0 years, with 55.31% women. Most of the patients belonged to the Medicare insurance group in both groups (76.59% and 74.26% in Group A and Group B, respectively) followed by private insurance (11.75% and 10.43% in Group A and Group B, respectively). Patients undergoing bronchoscopy were found to have fewer comorbidities as described by a CCI score of three or more (32.77% in Group A and 33.68% in Group B). Patients were evenly distributed in the income group, with most patients belonging to small metropolitan areas in Group A (61.28%) and large metropolitan areas in Group B (45.05%). Most of the patients had the procedure in large, teaching, and urban hospitals. The major comorbidities in both the population groups with and without bronchoscopy consisted of HTN (51% in Group A, 54.27% in Group B), dyslipidemia (41.21% in Group A, 37.81% in Group B), cigarette smoking (34.63 % in Group A, 35.73% in Group B), DM (27.78% in Group A, 29.47% in Group B), CHF (25.48% in Group A, 28.33% in Group B), GERD (25.40% in Group A, 22.60% in Group B), and CAD and CAD equivalents (22.65% in Group A, 22.72% in Group B).

**Figure 1:**
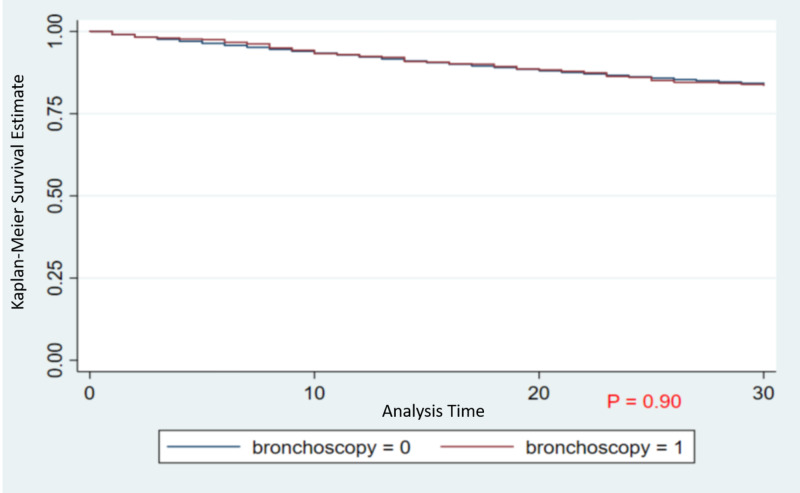
The Kaplan-Meier survival curve for the COPD populations undergoing bronchoscopy compared to those not undergoing bronchoscopy Abbreviation: COPD, chronic obstructive pulmonary disease

A total of 1,358 patients in Group A and 365,817 patients in Group B were discharged alive following IA. Figure 1 shows the Kaplan-Meier survival curve for the COPD populations undergoing bronchoscopy compared to those not undergoing bronchoscopy.

Two hundred fifteen patients were readmitted within 30 days (30-day all-cause readmission rate of 17.32%) in Group A and 58,070 (30-day all-cause readmission rate of 15.87%) in Group B. The mean time to bronchoscopy was 4.96 ± 9.18 days from admission. In our study, 8.6% and 1.3% of patients died during IA in Groups A and B, respectively.

For all patients undergoing bronchoscopy, the mean LOS during IA was 11.91 ± 20.21 days, which was higher than that of readmission (5.87 ± 5.48 days, P < .01). Mean total charge of IA for patients undergoing bronchoscopy ($107,358 for IA vs. $47,982 for readmission, P < .05) was higher than that of readmission, which also holds true for a mean total cost ($26,916 for IA vs. $12,374 for readmission, P < .05). The entire LOS for readmission in this group was 1,265 days leading to a total cost of $2.66 million.

However, for patients not undergoing bronchoscopy during IA (Group B), mean LOS was longer in readmissions (5.39 ± 5.51 days) than IAs (4.26 ± 4.27 days, P < .01). The mean total charge of readmission was higher than IA ($28,990 for IA vs. $40,469 for readmission, P < .01). A similar trend was noted for the mean total cost of IA vs. readmission in Group B ($8,137 for IA vs. $10,893 for readmission). The total LOS in Group B patients was 313,287 days, with a total cost of readmission of $628 million (Table 2).

**Table 2:** : Resource utilization Abbreviations: COPD, chronic obstructive pulmonary disease; LOS, length of stay

	COPD and Bronchoscopy	COPD without Bronchoscopy
Total admission	N = 1,358	N = 365,817
Discharged alive	1241	361,038
Readmitted	215	58,070
Mean LOS, days	0.00	0.00
Index admission	11.91 ± 20.21	4.26 ± 4.27
Readmission	5.87 ± 5.48	5.39 ± 5.51
Mean total charge ($)	0.00	0.00
Index admission	107,358	28,990
Readmission	47,982	40,469
Mean total cost ($)	0.00	0.00
Index admission	26,916	8,137
Readmission	12,374	10,893
Total LOS	1,265	313,287
Total cost of readmission	2.66 million	628 million
Total charge of readmission	10.3 million	2,330 million

The five most common causes of readmission in patients who underwent bronchoscopy were acute COPD exacerbation (32.36%), pneumonia (7.6%), sepsis (6.06%), acute on chronic respiratory failure (5.70%), and chronic obstructive asthma with acute exacerbation (5.45%). On the other hand, in Group B, acute COPD exacerbation (29.25%), acute on chronic respiratory failure (6.51%), pneumonia (6.28%), sepsis (5.27%), and acute bronchitis with COPD (3.5%) were the five most common causes of readmission (Table 3 and Table 4).

**Table 3:** Most common causes of readmission in group A Abbreviation: COPD, chronic obstructive pulmonary disease

Diagnosis	ICD-9 Code	COPD and Bronchoscopy
Acute COPD Exacerbation	491.21	32.36
Pneumonia	486	7.60
Septicemia	038.9	6.06
Acute or chronic respiratory failure	518.84	5.70
Chronic obstructive asthma with acute exacerbation	493.22	5.45
Orthostatic hypotension	458.0	3.13
Fluid overload	276.69	2.45
Abdominal pain	789.02	2.43
Atrial fibrillation	427.31	1.63
Tracheostomy complications	519.09	1.59

**Table 4:** Most common causes of readmission in group B Abbreviation: COPD, chronic obstructive pulmonary disease

Diagnosis	ICD-9 Code	COPD Without Bronchoscopy
Acute COPD exacerbation	491.21	29.25
Acute or chronic respiratory failure	518.84	6.51
Pneumonia	486	6.28
Septicemia	038.9	5.27
Acute bronchitis with COPD	49122	3.50
Acute respiratory failure	518.81	3.49
Acute asthma exacerbation	493.22	3.34
Acute on chronic diastolic heart failure	428.33	2.17
Atrial fibrillation	427.31	1.76
Acute or chronic systolic heart failure	42823	1.64

The independent predictors of readmission in Group A were pulmonary HTN (Hazard Ratio [HR] 2.35; 95% confidence interval [CI] 1.26-4.25; P < .01), adrenal insufficiency (HR 4.47; 95% CI 1.44-13.85; P = .01), and discharge to rehab (HR 3.17; 95% CI 1.05-9.52; P = .04).The remaining variables did not influence 30-day readmission rate significantly for patients undergoing bronchoscopy (Table 5).

**Table 5:** Predictors of readmission Abbreviations: AKI, acute kidney injury; CAD, coronary artery disease; CHF, congestive heart failure; CKD, chronic kidney disease; COPD, chronic obstructive pulmonary disease; GERD, gastroesophageal reflux disease; MI, myocardial infarction; PVD, peripheral vascular disease

	COPD and Bronchoscopy			COPD without Bronchoscopy		
Variable	Hazard Ratio	95% confidence interval	P value	Hazard Ratio	95% confidence interval	P value
Gender	0.65	0.42-1.01	.06	0.91	0.88-0.93	.00
Insurance						
Medicaid	1.41	0.73-2.73	.31	1.21	1.15-1.26	.00
Private	0.69	0.30-1.61	.39	0.75	0.71-0.79	.00
No insurance	3.01	0.56-16.19	.20	0.78	0.71-0.86	.00
Income						
$39,000-$47,999	0.92	0.58-1.47	.73	0.94	0.90-0.97	.00
$48,000-$62,999	1.07	0.56-2.04	.84	0.90	0.86-0.94	.00
≥ $63,000	1.28	0.61-2.66	.51	0.85	0.81-0.89	.00
Hospital setting						
Small metropolitan areas with less than one million residents	0.79	0.50-1.25	.32	0.90	0.86-0.94	.00
Micropolitan areas	0.65	0.15-2.84	.57	0.85	0.80-0.90	.00
Non-urban residual	2.99	0.42-21.25	.27	0.88	0.80-0.97	.01
Charlson comorbidity index	1.04	0.92-1.18	.50	1.08	1.06-1.09	.00
Bed size						
Medium	0.84	0.34-2.06	.71	1.03	0.98-1.08	.19
Large	1.22	0.55-2.72	.63	0.99	0.95-1.04	.80
Teaching	1.05	0.68-1.62	.81	0.97	0.93-1.01	.14
Hospital volume quintile						
2	1.20	0.48-2.99	.69	1.06	0.93-1.20	.38
3	1.09	0.45-2.65	.85	1.14	1.01-1.29	.04
4	0.86	0.37-2.02	.73	1.19	1.06-1.34	.00
5	0.99	0.48-2.06	.98	1.25	1.11-1.41	.00
Comorbidities						
Atrial fibrillation	1.42	0.86-2.36	.17	1.16	1.12-1.20	.00
AKI	1.35	0.72-2.53	.35	1.03	0.97-1.08	.35
Respiratory complications	1.60	0.94-2.72	.09			
Anemia	0.23	0.05-1.00	.05	1.06	0.99-1.13	.12
CAD and CAD Equivalent	1.32	0.84-2.06	.22	1.10	1.06-1.14	.00
Smoking	0.66	0.41-1.05	.08	0.94	0.91-0.97	.00
Prior stroke	0.96	0.42-2.17	.92	1.05	0.99-1.11	.12
Prior MI	1.17	0.49-2.77	.73	0.94	0.90-1.00	.03
GERD	1.19	0.76-1.87	.44	1.05	1.02-1.09	.00
Pulmonary hypertension	2.35	1.26-4.25	.00	1.03	0.98-1.08	.28
Hypertension	0.97	0.64-1.48	.89	0.97	0.94-1.00	.03
Obesity	1.16	0.71-1.88	.56	0.97	0.93-1.00	.08
Dyslipidemia	1.11	0.74-1.66	.62	0.89	0.86-0.92	.00
PVD	1.56	0.69-3.57	.28	0.97	0.92-1.03	.34
Diabetes	1.09	0.72-1.68	.68	1.04	1.00-1.07	.04
CHF	1.23	0.75-2.02	.41	1.18	1.14-1.22	.00
CKD	1.30	0.71-2.35	.40	0.93	0.88-0.99	.02
Adrenal Insufficiency	4.47	1.44-13.85	.01	1.28	0.98-1.67	.08
Discharge to Rehab	3.17	1.05-9.52	.04	0.99	0.80-1.22	.90
Length of stay	1.00	0.99-1.01	.62	1.01	1.01-1.02	.00

Independent predictor variables of admission in Group B were gender (women were at lower risk for readmission; HR 0.91; 95% CI 0.88-0.93; P < .01), type of insurance (Medicaid patients were more likely and privately insured patients were less likely to be readmitted as compared to Medicare), annual income (increased income was a negative predictor for readmissions), and Charlson comorbidity index with higher CCI related to increased readmission (HR 1.08; 95% CI 01.06-1.09; P < .01). Hospitals located in large metropolitan areas were associated with a higher chance of readmission compared to other settings. A positive relationship was also noted between hospital volume quintiles and the risk of readmission.

The medical comorbidities that significantly affected the readmission risk in Group B were atrial fibrillation (HR 1.16; 95% CI 1.12-1.20; P < .01), CAD and CAD equivalents (HR 1.10; 95% CI 1.06-1.14; P < .01), smoking (HR 0.94; 95% CI 0.91-0.97; P < .01), GERD (HR 1.05; 95% CI 1.02-1.05; P < .01), dyslipidemia (HR 0.89; 95% CI 0.0.86-0.92; P < .01), CHF (HR 1.18; 95% CI 1.14-1.22; P < .01), and CKD (HR 0.93; 95% CI 0.88-0.99; P = .02). Discharge disposition did not have any effect on readmissions in this group.

## Discussion

Our comprehensive analysis of the 2016 Nationwide Readmission Database (NRD) for 30-day all-cause readmission among COPD patients undergoing bronchoscopy (Group A) revealed the following findings: (1) all-cause 30-day readmission rate for patients undergoing bronchoscopy was 17.32%; (2) the most common causes of readmission were COPD exacerbation, pneumonia, and sepsis; (3) a total of 1,265 days and $2.66 million were lost to readmission following bronchoscopy; (4) significant predictors of readmission in this study are pulmonary hypertension, adrenal insufficiency and discharge to rehabilitation; (5) there was a statistically significant difference in the total cost and total charge between index hospitalization and readmission.

Analysis of data from COPD patients without bronchoscopy (Group B) showed the following: (1) 15.87% of patients were readmitted within 30 days following discharge in Group B; (2) the most common causes of readmission were COPD exacerbation, acute on chronic respiratory failure, and pneumonia; (3) the total LOS was 313,287 days, with the total cost of readmission as $2.33 billion; (4) there was a statistically significant difference in the total cost and total charge between index hospitalization and readmission, similar to Group A.

According to various studies, the overall mortality rate following bronchoscopy was approximately 0.013% to 0.02% [6,7]. Respiratory complications following bronchoscopy are found more often in patients with severe to very severe COPD (22%) compared with patients without COPD (6%) [8].

The risk of readmission in COPD patients is high. After discharge, 10% to 20% of COPD patients are readmitted within 30 days, which correlates to our readmission rate (17.32% in Group A and 15.87% in Group B) [9,10]. The major causes of readmission following index COPD admission are COPD exacerbation, CHF, pneumonia, and skilled nursing facility patients [9]. Compared with IAs, COPD patients have longer LOS during readmission [9]. These findings were not so different as compared to this study where COPD exacerbation and pneumonia were among the most common readmission diagnoses in both Group A and Group B. Patients who are readmitted following a COPD hospitalization are at greater risk of mortality and have worse outcomes relative to patients who are not readmitted [11].

For patients with bronchoscopy, the readmission rate was slightly higher than for the patients not undergoing bronchoscopy (17.32% vs. 15.87%). The mean LOS for both IA and readmission was longer in Group A patients (COPD with bronchoscopy) as compared to Group B patients (COPD without bronchoscopy). The difference could be attributed to possible higher disease severity/higher comorbidity burden in COPD patients who had undergone bronchoscopy requiring prolonged hospitalization and treatment as compared to patients not undergoing bronchoscopy or the possible complications associated with bronchoscopy due to localized microtrauma causing increased airway inflammation leading to a prolonged hospital stay.

We found significant use of resources with readmission within 30 days. Readmission could have prevented LOS of 1,265 hospital days, and $10.3 million costs in Group A and 313,287 hospital days and $2.33 billion costs in Group B. This is particularly important in the context of modern-day care where cost-effective and affordable healthcare is emphasized. Over the three years of the Hospital Readmission Reduction Program (2012 to 2015), the Center for Medicare and Medicaid dollar estimates of penalties increased from $290 million to $428 million [12].

The mortality rate was higher in the bronchoscopy group as compared to the COPD only group (8.6% vs. 1.3%, respectively). Due to the limitations of the database, specific mortality was not available. However, from the literature review, we noted the majority of complications occur in patients with high levels of comorbid disease undergoing more extensive therapeutic interventions. Over the years, the rate of complications associated with bronchoscopy has gone down significantly due to technological advances and improved skills of physicians and technicians. Overall, flexible bronchoscopy is a safe and effective procedure for the diagnosis and treatment of airway and pulmonary pathology [13].

Figure 1 shows the Kaplan-Meier survival curve for the COPD populations undergoing bronchoscopy versus not undergoing bronchoscopy. Bronchoscopy during IA is associated with decreased LOS in subsequent visits and thereby decreased cost of readmission. With the shorter duration of subsequent 30-day readmission, resources could be saved, and reimbursement can be increased, which will add to the financial benefits of such interventions.

The 10 leading causes for readmission after the IA in decreasing frequency are shown in Table 3 and Table 4. The literature revealed that the major causes of readmission following index COPD admission are COPD, CHF, pneumonia, and skilled nursing facility patients [9]. We found that COPD was the most common cause in both groups, accounting for 32.36% and 29.25% readmissions in Groups A and B, respectively. CHF, which clinically mimics COPD, explained 2.45% and 3.8% of all readmissions in Groups A and B, respectively. A wide array of reasons was responsible for readmission; however, after the first five major diagnoses, the remaining codes had individual frequencies < 5%. Stratification showed that only 52.7% of readmissions in Group A and 52.4% readmissions in Group B were due to respiratory-related causes. Group A had a higher rate of readmission from patients initially discharged to rehab than in those with other discharge dispositions. This difference was not significant for Group B.

Shah et al. have reported in their study that COPD was the most common cause of readmission; CHF was the second cause of all readmission. Subsequent causes of admission had a < 5% prevalence [9]. The findings reported in our study were similar to this study.

In our study, significant readmission predictors for patients undergoing bronchoscopy are pulmonary hypertension, adrenal insufficiency, and discharge disposition to rehab.

In Group B, readmission predictors were gender, insurance, and CCI. Patients not undergoing bronchoscopy had an overall sicker population with a high burden of multiple comorbidities (CCI ≥ 2), which could discourage the physicians from considering bronchoscopy in this group. A high CCI score explains prolonged LOS during readmission, probably due to frailty in this patient population. In Group B, with each point increase in the CCI, there are 8% odds of increased risk for readmission.

Sharif et al. reported that among COPD patients, 30-day readmission was more common in patients with a history of CHF, lung cancer, anxiety, obesity, depression, osteoporosis, CKD, DM, HTN, and obstructive sleep apnea compared with those who did not have these comorbidities. There was a stepwise increase in rates of 30-day readmission with an increasing number of comorbid medical conditions. These findings are not so different from the outcomes observed in the present study [10].

In our study, patient-related variables were often the primary predictor of readmission. Links have been identified between some comorbidities and COPD based on the available evidence, such as its association with cardiovascular disease after adjusting for confounders, with much focus on the significant role of systemic inflammation, which is a characteristic feature of many chronic medical diseases [14-17].

During discharge, care can be taken to identify patients with these factors and label them as high risk for readmission. The intervention aimed to track those patients can reduce 30-day readmission. Interventions that have been successful are case management, patient education with written action plans, pulmonary rehabilitation (ideally started within 30 days of discharge), and smoking cessation programs [18]. Technological advances linking outpatients with healthcare professionals (e.g., respiratory therapists, nurses, dietitians, pharmacists, physicians) have progressed faster than validation studies demonstrating improved outcomes. Examples include direct observation of inhaler technique by video-audio links (e.g., FaceTime, Skype), tablet- and/or smartphone-based applications (e.g., COPD Navigator), digital diaries for symptom tracking, home pulse oximetry, and peak expiratory flow. There is increasing recognition of the essential role of respiratory therapists in preventing readmission following COPD exacerbation [19].

Bronchoscopy can be considered as an intervention that could be used as a diagnostic and therapeutic intervention in certain COPD patients where it is indicated, leading to a shorter course of subsequent readmission and overall decreased healthcare resource utilization.

Our study has several limitations. First, it is an administrative database where coding practices are not uniform among different hospitals. Data regarding medication and laboratory parameters are not available in the database. There is no information regarding the severity of COPD, indication for the procedure, or imaging results. Patients readmitted to another state cannot be traced in the NRD database, but this proportion should be low. Mortality data cannot be calculated outside the hospital after discharge because mortality during travel, in the emergency room, or at home cannot be accounted to identify the influence of death on readmission rates. Our information is presented in an extensive database minimizing the chances of beta error. Similarly, the population included in the study came from different strata of hospitals, comprising different insurance statuses, includes population from large, medium, and small hospitals, and teaching vs. non-teaching hospitals, making results generalizable for patients admitted for COPD undergoing bronchoscopy. Because it uses an enormous sample size, it can also give insight about uncommon risk factors, which is a limitation of single-center cohort studies.

## Conclusions

The readmission rate in COPD patients is very high and is impacted by a variety of social, personal, and medical factors. Patients with multiple medical comorbidities have a higher risk of readmission. Early interventions and close follow-up in high-risk patients could help prevent these readmissions and decrease healthcare resource utilization. Patients undergoing bronchoscopy have a slightly higher rate of 30-day readmission as compared to patients not undergoing bronchoscopy; however, the LOS is shorter during readmission in this group.
